# Altered Intestinal Production of Volatile Fatty Acids in Dogs Triggered by Lactulose and Psyllium Treatment

**DOI:** 10.3390/vetsci9050206

**Published:** 2022-04-23

**Authors:** Máté Mackei, Rebeka Talabér, Linda Müller, Ágnes Sterczer, Hedvig Fébel, Zsuzsanna Neogrády, Gábor Mátis

**Affiliations:** 1 Division of Biochemistry, Department of Physiology and Biochemistry, University of Veterinary Medicine, István Street 2, H-1078 Budapest, Hungary; talaber.rebeka.reka@student.univet.hu (R.T.); neogrady.zsuzsanna@univet.hu (Z.N.); matis.gabor@univet.hu (G.M.); 2 Department of Obstetrics and Food Animal Medicine Clinic, University of Veterinary Medicine, István Street 2, H-1078 Budapest, Hungary; muller.linda@univet.hu; 3 Department and Clinic of Internal Medicine, University of Veterinary Medicine, István Street 2, H-1078 Budapest, Hungary; sterczer.agnes@univet.hu; 4 Nutrition Physiology Research Group, Institute of Physiology and Nutrition, Kaposvár Campus, Hungarian University of Agriculture and Life Sciences, Gesztenyés Street 1, H-2053 Herceghalom, Hungary; hullarne.febel.hedvig@uni-mate.hu

**Keywords:** prebiotics, canine nutrition, gut health, volatile fatty acids, intestinal microbial fermentation

## Abstract

The intestinal microbiome of dogs can be influenced by a number of factors such as non-starch polysaccharides as well as some non-digestible oligo- and disaccharides. These molecules are only decomposed by intestinal anaerobic microbial fermentation, resulting in the formation of volatile fatty acids (VFAs), which play a central role in maintaining the balance of the intestinal flora and affecting the health status of the host organism. In the present study, the effects of lactulose and psyllium husk (*Plantago ovata*) were investigated regarding their influence on concentrations of various VFAs produced by the canine intestinal microbiome. Thirty dogs were kept on a standard diet for 15 days, during which time half of the animals received oral lactulose once a day, while the other group was given a psyllium-supplemented diet (in 0.67 and in 0.2 g/kg body weight concentrations, respectively). On days 0, 5, 10 and 15 of the experiment, feces were sampled from the rectum, and the concentration of each VFA was determined by GC-MS (gas chromatography–mass spectrometry). Lactulose administration caused a significant increase in the total VFA concentration of the feces on days 10 and 15 of the experiment (*p* = 0.035 and *p* < 0.001, respectively); however, in the case of psyllium supplementation, the concentration of VFAs showed a significant elevation only on day 15 (*p* = 0.003). Concentrations of acetate and propionate increased significantly on days 5, 10 and 15 after lactulose treatment (*p* = 0.044, *p* = 0.048 and *p* < 0.001, respectively). Following psyllium administration, intestinal acetate, propionate and n-butyrate production were stimulated on day 15, as indicated by the fecal VFA levels (*p* = 0.002, *p* = 0.035 and *p* = 0.02, respectively). It can be concluded that both lactulose and psyllium are suitable for enhancing the synthesis of VFAs in the intestines of dogs. Increased acetate and propionate concentrations were observed following the administration of both supplements; however, elevated n-butyrate production was found only after psyllium treatment, suggesting that the applied prebiotics may exert slightly different effects in the hindgut of dogs. These findings can be also of great importance regarding the treatment and management of patients suffering from intestinal disorders as well as hepatic encephalopathy due to portosystemic shunt.

## 1. Introduction

Short chain or volatile fatty acids (VFAs) produced by the bacteria that are members of the physiologic colonic ecosystem play a central role in maintaining gut health and function [1]. VFA molecules consist of one to seven carbon atoms, containing either straight or branched chain compounds. They are formed as products of anaerobic microbial fermentation, mainly by the breakdown of plant compounds, such as cellulose, resistant starch (RS), non-starch polysaccharides (NSP) and oligosaccharides [2,3,4]. The three main intestinal VFAs are acetic acid, propionic acid and n-butyric acid. These organic acids account for more than 95% of the VFA content [5]. Since canine species do not produce non-digestible carbohydrate-degrading enzymes, intestinal anaerobic microbial fermentation and VFA synthesis are of great importance [6]. Considering that various VFA molecules have specific effects on gut integrity, energy metabolism, immune response and the intestinal microbiome, studies focusing on the interplay of feed additives and VFA production are of great interest [7,8,9].

Lactulose, a synthetic isomeric form of lactose, contains a galactose linked to a fructose molecule by a ß-1,4 bond. This disaccharide is normally only digested by intestinal bacteria in the large intestines to produce lactate, acetate, methane and hydrogen. Lactulose has been used in human medicine since the 1960s [10]. Its main mechanism of action is to reduce the intestinal synthesis and absorption of ammonia [11]. Colonic metabolism of lactulose induces a laxative effect by increasing intraluminal gas formation and osmolality, leading to a decrease in transit time as well as in lower intraluminal pH [12]. Lactulose also promotes the bacterial uptake of ammonia in the colon, which uses it as a nitrogen source for protein synthesis [11]. Lowering gut pH facilitates this process, promoting the conversion of ammonia (NH_3_) produced by gut bacteria into ammonium ion (NH_4_^+^), which has a significantly lower absorption rate from the gut, leading to decreased workload for the liver as well as less intense urinary nitrogen excretion [13]. This support of the hepatic detoxification capacity is also one of the main reasons why lactulose can be applied as an efficient feed additive in individuals suffering from portosystemic shunt (PSS) [11,13].

The prolonged bacterial degradation of psyllium husk (a plantain seed coat of *Plantago ovata*, hereafter psyllium) leading to the production of large amounts of VFAs has been already demonstrated in vitro as well as in vivo, but there are no exact literature data on its effect regarding VFA production and the possibly altered microbiota in the canine colon [6]. Psyllium mostly consists of highly branched and gel-forming arabinoxylan as a soluble NSP, a polymer rich in arabinose and xylose [14]. As its breakdown in the proximal intestinal tract is limited, psyllium has high prebiotic potential [14]. Dietary fibers presented in psyllium can aid in the normalization of colonic motility and transit time, as well as support normal gut microbiota development and provide energy for the colonocytes [7]. Soluble fibers can be added to a normal diet to enhance feces consistency since they have high water-holding capabilities, serving as a hydrocolloid source, containing a high amount of hydroxyl groups that increase water binding capacity [15,16]. The abovementioned effects result in more viscous intestinal content which can be beneficial in the treatment of diarrheal diseases, as described also in dogs [17]. Psyllium can also have an impact on the composition of the large intestinal microbiota and increase the relative weight of the colon as well as the surface area, hence enhancing absorptive capacity [18]. Due to its flavonoid and phenol content, psyllium has antioxidant effects, while the essential fatty acids (omega-3 and omega-6), as well as sulfur-containing amino acids and bioactive amines make it a promising food and feed supplement [19]. It is also frequently used in human nutrition to promote weight control, regulate glucose homeostasis in diabetics and reduce serum lipid levels in hyperlipidemia [19]. On the other hand, based on its high fiber content, by the stimulation of intestinal VFA synthesis, it may be also beneficial in humans and animals suffering from kidney or liver failure, PSS and hepatic encephalopathy (HE) [20].

The aim of our study was to investigate the effect of lactulose and psyllium husk on the bacterial fermentation in the intestinal tract of dogs by assessing the VFA concentrations in freshly collected fecal samples. These orally administered prebiotics may be beneficial substrates for the canine gut microbiome, producing VFAs by bacterial breakdown. Lactulose and psyllium may thus help to provide and maintain the balance of the intestinal flora and the proliferation of beneficial intestinal bacteria [12,16]. Produced VFAs may have several positive physiological effects on the intestinal epithelium and, when absorbed, on extraintestinal tissues such as the liver, the kidneys and the brain, as well [21,22,23,24].

## 2. Materials and Methods

### 2.1. Animals, Treatments and Samplings

The study was performed using 30 healthy beagle dogs at the Aurigon Toxicological Research Centre Ltd. (Dunakeszi, Hungary).

Housing, feeding and treatment of the animals were conducted in strict accordance with the national and international laws as well as with the institutional guidelines. Experimental procedures were approved by the Government Office of Pest County, Food Chain Safety, Animal Health, Plant and Soil Protection Department (project permit PE/EA/558-5/2019, approval date: 29 April 2019). Animals were housed in pairs in a compacted box with a floor area of 4.8 m^2^. The temperature in the animal house was continuously monitored and set to 20 °C with 60% relative humidity. Artificial lighting was used, with a 12:12 h dark/light cycle. The kennels were cleaned daily, and the health and welfare of the animals was also monitored daily. The animals were provided with drinking water ad libitum and were given dry food once a day in the morning (Ecopet Natural Adult Medium, Farmina Pet Foods Ltd., Nola, Italy). An important prerequisite for participation in the trial was that none of the animals should have been on any medication or supplementation to avoid any possible alteration in the status of the intestinal flora. In addition, the age and sex of the dogs were taken into account, and accordingly, animals over 1 year of age were selected in a 2:1 male to female sex ratio. Age, sex and body weight of the selected animals are indicated in Table S1.

Blood samples were taken into tubes containing ethylenediaminetetraacetic acid (EDTA) or heparin from *v. cephalica antebrachii* of the dogs one week before the start of the experiment. Following centrifugation, plasma samples were immediately stored at −20 °C. Hematological and biochemical analyses (Tables S2 and S3, respectively) of the obtained whole blood as well as plasma were carried out to check the overall health status of the animals and to confirm no deviation from the reference values for healthy animals.

The first group (*n* = 15) was given oral lactulose supplementation once a day before feeding for 15 days. Animals in the second group (*n* = 15) were given psyllium, once daily at the same time as the lactulose treatment. Lactulose supplementation was given at a dose of 1 mL/kg body weight per day (670 mg/mL lactulose), while psyllium was given in 0.2 g/kg body weight concentration. Duration of the treatments, along with the applied lactulose and psyllium dosage were determined based on previous dog-related studies carried out by other research groups [11,17,18,25,26]. Fecal samples were taken manually by palpating the *ampulla recti* on days 0, 5, 10 and 15 before treatments. Samples were shock-frozen in dry ice and stored at −80 °C until further measurements.

### 2.2. Laboratory Analyses

Whole blood and blood plasma samples collected before the start of the experiment were analyzed with a Sysmex XT-2000i analyzer (Sysmex, Norderstedt, Germany) for hematological parameters, and with a Konelab 60i chemical analyzer (Thermo Fisher Scientific, Waltham, MA, USA) for biochemical parameters. Since no values significantly deviated from the reference range, no selected animals had to be excluded from the experiment (Tables S2 and S3).

VFAs were separated by gas chromatography–mass spectrometry (GC-MS) and their concentrations were determined. Measurements were performed using 0.8 g feces to which 8 mL of distilled water, 0.135 mL of 0.2 M sodium hydroxide (NaOH) solution and 0.7 mL of 2-ethylbutyric acid solution (200.4 mg/100 mL as internal standard) were added. Following homogenization and centrifugation, 2.1 mL supernatant was transferred to a test tube to which 0.25 mL of metaphosphoric acid solution (8 g metaphosphoric acid +20 mL water, 28.57 m/m%) was added, vortexed and finally diluted 1:1 with 4.25 m/m% metaphosphoric acid solution. VFA content was determined using GCMS-QP2010 SE (Shimadzu Co., Kyoto, Japan). The temperature program was: 75–175 °C with a heating rate of 11 °C/min, 175 °C for 1 min. Column: Zebron ZB-WAX 30 m × 0.25 mm × 0.25 μm (Phenomenex, Torrance, CA, USA). Injected volume was 0.5 μL, while injection temperature was set to 235 °C, applying a splitless injection method. Helium gas was used as carrier, and flow control mode was set to linear flow at 119.9 kPa. The total flow was set to 77.1 mL/min; for this, the column flow was 1.81 mL/min and the linear velocity was 49.4 cm/s. The purge flow was set to 3 mL/min.

### 2.3. Statistics

Data analysis was performed using R 3.5.3. software (GNU General Public License, Free Software Foundation, Boston, MA, USA). Samples were analyzed using paired *t*-tests, considering day 0 (fecal sample before starting the treatment) as the control. Normal distribution and homogeneity of variance were checked using the Shapiro–Wilk test and Levene’s test, respectively. Differences were considered significant at *p* < 0.05.

## 3. Results

**Figure 1:**
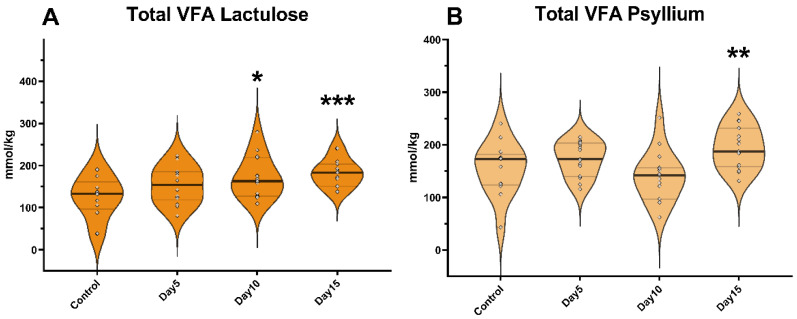
Violin plots show the total volatile fatty acid (VFA) concentration in the fecal samples following lactulose (**A**) and psyllium (**B**) treatment measured by GC-MS method. Data are visualized using violin plots. Concentrations of single samples are plotted as gray dots; black line refers to the median and gray lines refer to first and third quartiles. * *p* < 0.05, ** *p* < 0.01, *** *p* < 0.001.

According to our results, the administration of lactulose did not cause a significant change in the total VFA concentration of the feces on day 5 of the experiment, which was also observed in the case of psyllium administration. On the other hand, the addition of lactulose on day 10 of the experiment significantly increased total VFA content (*p* = 0.035), while psyllium administration did not cause a significant increase. On day 15 of the experiment, the administration of both lactulose and psyllium significantly elevated total VFA concentration (*p* < 0.001, *p* = 0.003, respectively; Figure 1).

**Figure 2:**
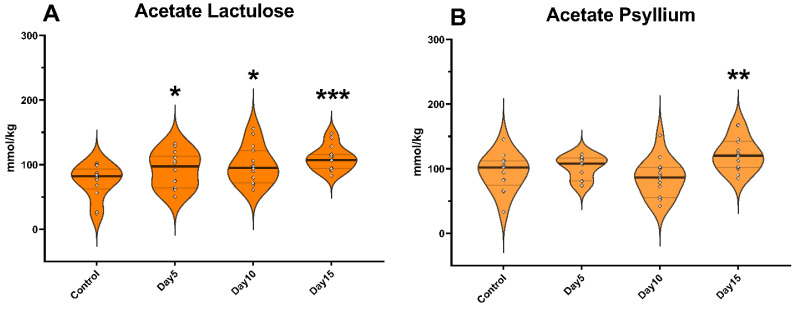
Violin plots show the acetate concentration in the fecal samples following lactulose (**A**) and psyllium (**B**) treatment measured by GC-MS method. Data are visualized using violin plots. Concentrations of single samples are plotted as gray dots; black line refers to the median and gray lines refer to first and third quartiles. * *p* < 0.05, ** *p* < 0.01, *** *p* < 0.001.

The concentration of acetate significantly increased following lactulose administration on day 5 of the experiment (*p* = 0.044). However, no significant increase was observed in the case of psyllium supplementation. On day 10, lactulose increased the amount of acetate (*p* = 0.048), while the administration of psyllium did not result in a significant change. On day 15 of the study, the amount of acetate was increased by both lactulose (*p* < 0.001) and psyllium (*p* = 0.002) administration compared to day 0 samples (Figure 2).

**Figure 3:**
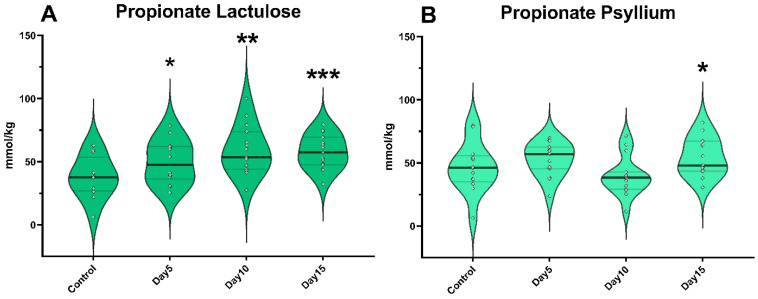
Violin plots show the propionate concentration in the fecal samples following lactulose (**A**) and psyllium (**B**) treatment measured by GC-MS method. Data are visualized using violin plots. Concentrations of single samples are plotted as gray dots; black line refers to the median and gray lines refer to first and third quartiles. * *p* < 0.05, ** *p* < 0.01, *** *p* < 0.001.

The amount of propionate was significantly higher (*p* = 0.041) following lactulose administration on day 5; however, psyllium did not cause a significant change in propionate concentration at this time. On day 10 of the experiment, lactulose administration significantly increased propionate concentration (*p* = 0.008). Administration of psyllium did not cause a significant change in propionate concentration on day 10. On the other hand, on day 15, the addition of both lactulose (*p* < 0.001) and psyllium (*p* = 0.035) resulted in a significant increase in propionate concentration (Figure 3).

**Figure 4:**
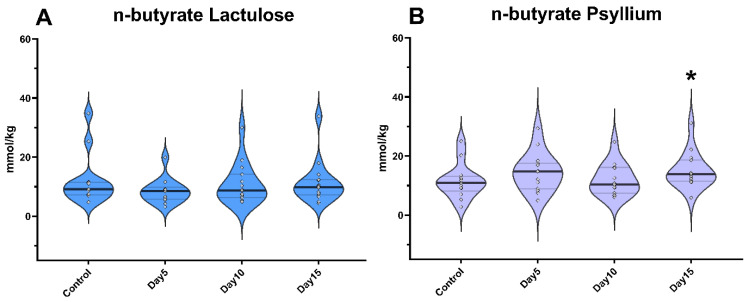
Violin plots show the n-butyrate concentration in the fecal samples following lactulose (**A**) and psyllium (**B**) treatment measured by GC-MS method. Data are visualized using violin plots. Concentrations of single samples are plotted as gray dots; black line refers to the median and gray lines refer to first and third quartiles. * *p* < 0.05.

On days 5 and 10 of the experiment, neither lactulose nor psyllium administration resulted in a significant change in n-butyrate levels. On day 15, lactulose did not significantly increase n-butyrate concentrations, but psyllium administration resulted in a significant elevation (*p* = 0.02; Figure 4).

No significant difference was observed in the concentration of isobutyrate in both the lactulose- and psyllium-treated groups (Figure S1). Regarding isovalerate and n-valerate content of feces samples, there was also no change observed in correlation with the treatments (Figures S2 and S3).

The proportions of the amount of VFAs compared to each other are expressed as mol%. These results of lactulose treatment were plotted using a pie chart. No significant change in the molar ratio results was found in the psyllium groups, except a slight increase in the percentage of isobutyrate on day 10 (*p* = 0.014).

**Figure 5:**
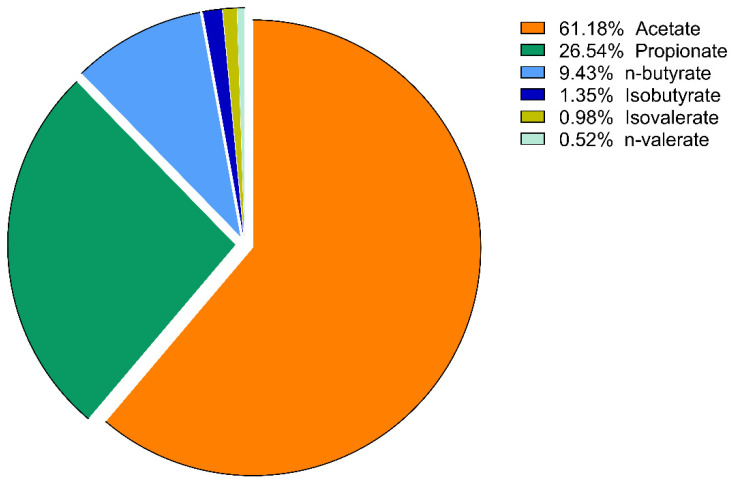
Volatile fatty acid profile of fecal samples of the lactulose group on day 0 of the experiment measured by GC-MS method. Data are expressed as mol%.

**Figure 6:**
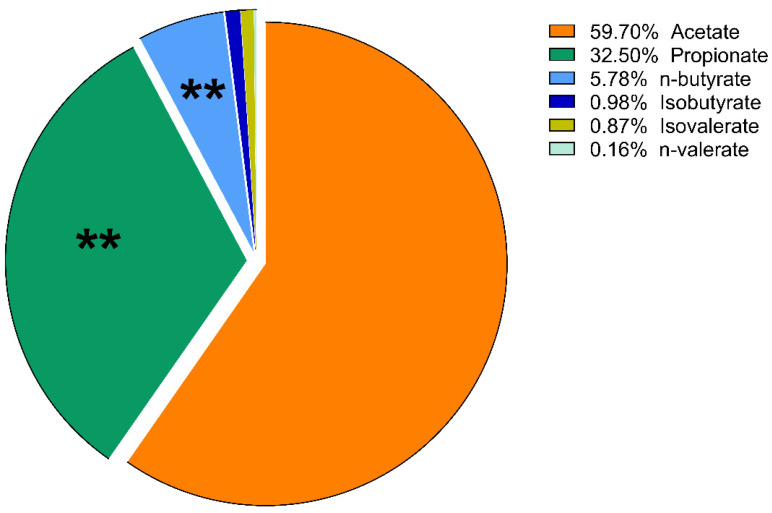
Volatile fatty acid profile of fecal samples of the lactulose group on day 5 of the experiment measured by GC-MS method. Data are expressed as mol%. ** *p* < 0.01.

**Figure 7:**
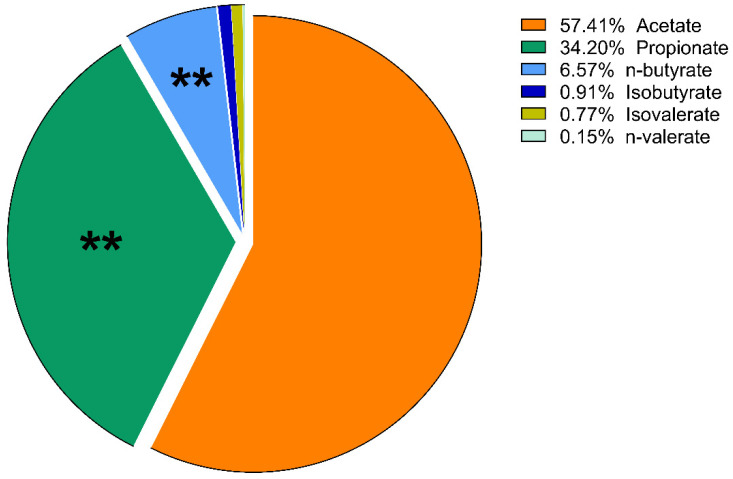
Volatile fatty acid profile of fecal samples of the lactulose group on day 10 of the experiment measured by GC-MS method. Data are expressed as mol%. ** *p* < 0.01.

**Figure 8:**
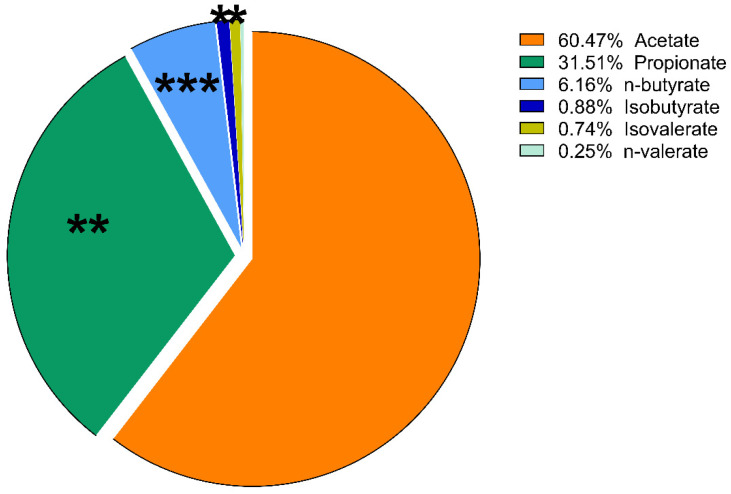
Volatile fatty acid profile of fecal samples of the lactulose group on day 15 of the experiment measured by GC-MS method. Data are expressed as mol%. * *p* < 0.05, ** *p* < 0.01, *** *p* < 0.01.

On day 5, there was a significant increase in the ratio of propionate in the lactulose group (*p* = 0.008; Figure 5 and Figure 6). At the same time, there was a significant decrease in n-butyrate proportion (*p* = 0.002) as the effect of lactulose administration. On day 10, there was also a significant increase in propionate ratio (*p* = 0.003), while the percentage of n-butyrate (*p* = 0.016) was significantly decreased compared to day 0 (Figure 7). On day 15, the molar ratio of propionate (*p* = 0.009) showed a significant increase, while the value of n-butyrate, isobutyrate acid and isovalerate decreased significantly with lactulose administration (*p* < 0.001; *p* = 0.017; *p* = 0.012, respectively; Figure 8).

## 4. Discussion

The application of prebiotics recently became a prominent research topic in the field of human and animal nutrition, including canine species. Certain feed additives can improve and stabilize the composition of the intestinal microbiota, reducing the presence of pathogens as well as toxins [27]. In addition, prebiotics may lead to enhanced immune function and can be used safely as a secondary treatment of diseases, such as certain bacterial intestinal infections, constipation, kidney or liver diseases, especially hepatic encephalopathy caused by PSS, explaining the increasing interest in the application of fiber and prebiotics such as lactulose and psyllium in animal feed [11,28,29,30,31,32,33].

Several studies have shown that VFAs derived from the microbial fermentation of carbohydrates are a source of energy that is beneficial to the host organism [6,7,8]. Colonocytes can effectively use n-butyrate as a substrate, covering their energy demand, while liver cells are able to metabolize both propionate and n-butyrate. On the other hand, peripheral tissues can mainly utilize acetate [34].

Energy amount derived from microbial fermentation is estimated to provide 2–7% of the maintenance energy requirements of an adult dog [35,36]. In addition, VFAs have anti-inflammatory effects because of increased anti-inflammatory cytokine production (e.g., interleukin (IL)-10 and transforming growth factor (TGF)ß), while decreasing the release of pro-inflammatory cytokines (e.g., IL-6, IL-8 and tumor necrosis factor (TNF)α) and activating certain transcription factors such as Foxp3, which are also involved in the regulation of inflammation [37]. These organic acids have also been shown to have selective antibacterial effects [38].

Their presence is beneficial not only for the host, but also for the microbiome that synthesizes them, as their production provides a relatively acidic environment in the gut, preventing the overgrowth of pH-sensitive pathogenic bacteria such as species belonging to the *Enterobacteriaceae* and *Clostridia* families [39]. The decrease in pH also affects the intestinal absorption of certain substances [13]. In terms of pH reduction caused by the acidic character of VFAs, ammonia is converted to ammonium ions, which are absorbed in a lesser amount from the intestines [40]. This may also be beneficial for the host organism, especially in individuals suffering from hepatic encephalopathy due to PSS or liver failure [40].

In the present study, changes in intestinal VFA concentration in dog fecal samples were investigated following oral supplementation of two prebiotics, lactulose and psyllium. The quantitative changes in total and individual VFAs on days 5, 10 and 15 of the experiment were compared to day 0.

The results of our study showed that lactulose significantly increased total VFA content more rapidly than psyllium. This may be due to the fact that the breakdown of lactulose as a disaccharide requires the induction of a single enzyme, in contrast to the complex oligo- and polysaccharide components of psyllium, where the breakdown requires the induction of the coordinated action of several enzymes [41,42,43]. Accordingly, microbial adaptation presumably also requires more time and more coordinated stimulation during psyllium fermentation, which may be reflected in the time shift that has been observed between the two treatments. According to previous dog-related studies, lactulose application was associated with a significant increase in Firmicutes and Actinobacteria (predominantly *Veillonellaceae* and *Bifidobacteriaceae*), and a decrease in Bacteroidetes and Fusobacteria (*Bacteroidaceae* and *Fusobacteriaceae*) already following a relatively short, 2-week-long supplementation [26]. These microbiota-associated changes may be presumably in the background of the altered VFA production as well, described in our study. Furthermore, another reason for the increased VFA concentrations may be their decreased luminal absorption. However, additional studies are necessary to clarify this question.

Acetate is the most abundant VFA in the colon and accounts for more than half of all VFAs detected in the feces. Its quantitative variation is therefore of indicative value [44]. Most enteric bacteria produce acetate as a result of carbohydrate fermentation, and it is mainly metabolized by peripheral tissues and is therefore present in relatively high amounts in the circulatory system [45]. Depending on the tissues and the metabolic state of the organism, in the form of acetyl-CoA, it can be involved in the citric acid cycle or as a substrate in the fatty acid synthesis [46].

The concentration of acetate was quickly and significantly increased by lactulose, while psyllium administration caused a significant increase only later, on day 15 of the trial. In correlation with our results, in a human study, psyllium supplementation increased the fecal acetate concentration along with the amount of both succinate-specific utilizer *Phascolarctobacterium* and acetate metabolizing *Faecalibacterium* species [15]. Furthermore, according to other studies, increased fiber content of feed resulted in intensely elevated acetogenic bacterial content such as *Ruminococcus*, *Phascolarctobacteria*, *Christensenellaceae* and *Ruminococcacea* species in dogs [47].

Considering our results, it can be stated that lactulose was a more sufficient supplement for rapidly increasing the concentration of acetate, also in the sense that it produced a significant increase already after 5 days, and that it increased the amount of acetate throughout the whole duration of the experiment. On the other hand, it is also important to mention that antimicrobial and anti-inflammatory activity of acetate are of less importance compared to propionate and n-butyrate [48].

Propionate, along with acetate, may play a role in the regulation of gluconeogenesis [49]. The first is also well known as a starting molecule for gluconeogenesis in ruminants and strict carnivores such as feline species, but this effect has also been observed in dogs [50]. Furthermore, propionate is a highly potent immunomodulatory and anti-inflammatory molecule [36], which can be used in human and animal nutrition due to its selective antimicrobial activity [51,52]. When used as a therapeutic supplement, it also reduced the severity of colitis in mouse models [37]. It has been also reported that propionate may be effective in reducing the number of *Salmonella*, *Campylobacter* and *Escherichia coli* species in the intestines of various species [51,52].

Lactulose resulted in a significant quantitative increase in propionate concentrations in all cases, while psyllium administration lead to a significant increase in propionate levels only on day 15 of the experiment. Furthermore, as mentioned previously, psyllium supplementation in humans increased the number of *Phascolarctobacterium* species which are involved in propionate synthesis [15]. *Phascolarctobacteria* were also described to have a positive correlation with the mood of humans and exerted beneficial effects on the host, nonalcoholic fatty liver models in rats [53]. Our results therefore suggest that both lactulose and psyllium were capable of increasing propionate concentrations, but similarly to the results regarding total VFA and acetate content, lactulose application resulted in increased propionate concentration already after 5 days. It was also found by other research groups that in dogs suffering from chronic enteritis, intestinal VFA, acetate and propionate concentrations were significantly reduced compared to healthy controls [48]. We obtained inversed results measuring the abovementioned parameters after administration of lactulose and psyllium. This may suggest that the mentioned parameters are typically altered during the adaptation of the bacterial flora in dogs, either negatively or positively affecting the health of the animal. According to our results, lactulose as well as psyllium may be potent agents in the normalization of VFA levels in dogs suffering from chronic enteritis. However, further studies could be of great importance to determine whether lactulose acts only as an energy-providing substrate, accelerating the growth and metabolism of acetate- and propionate-producing bacteria, or if the molecule and its metabolites may have other regulatory roles. Furthermore, the roles of lactulose and psyllium in the treatment of HE and PSS have also become an area of high interest nowadays, since these agents are able to effectively reduce the availability and production of nitrogenous waste products in the gastrointestinal tract, as described previously [54,55]. Supporting this hypothesis, decreased intestinal nitrogen absorption, due to the effect of lactulose treatment, has been described in dogs, too, leading to decreased workload for the liver.

The amount of n-butyrate, which is not degraded by the intestinal epithelial cells, enters the portal circulation and is delivered to the liver, where it can be involved in the β-oxidation and ketogenesis of hepatocytes. Butyrate is considered to have the highest biological activity of all VFAs [56]. It is widely used as an alternative feed additive, due to its positive effects on weight gain and feed conversion [57,58]. It is also necessary for the proper development of GALT (gut-associated lymphoid tissue) [59]. It enhances the expression of the tight junction proteins such as occludin and cingulin, responsible for cellular adhesion, thus improving gut barrier function [60]. Butyrate is also known to have a selective antimicrobial effect, whereby bacterial species sensitive to elevated n-butyrate concentrations, such as enterotoxigenic *E. coli* strains, *Salmonella* and *Clostridium* species, are eliminated, while the beneficial microflora is not impaired [61,62]. Part of n-butyrate is metabolized by liver cells, but some amount that is not broken down during metabolism may have epigenetic effects. It is able to alter the structure of the chromatin pool by changing the rate of acetylation of histone proteins and by modifying the methylation of DNA, i.e., the expression of certain genes [63,64].

According to our results, it can be also concluded that only psyllium was able to increase the concentration of n-butyrate, which can be in correlation with the shift in the canine intestinal microbiota towards *Faecalibacterium, Christensenellaceae, Oscillospira* and *Ruminococcus* spp. as the effect of fiber-rich diet, described in previous studies, since these bacteria are able to effectively convert sugars, acetate and other substrates into n-butyrate [15,47]. On the other hand, according to recent studies, the composition of the intestinal microbiota of healthy humans was less altered by psyllium administration than that of constipated people [15,65]. This is also in line with further findings regarding psyllium supplementation, as well as the general idea that the microbial population of healthy persons is more resistant to environmental changes [66].

Our findings show that psyllium was able to increase only the proportion of isobutyrate significantly on day 10 of the investigation. Isobutyrate may also serve as an alternative source for energy production in the colonocytes; however, it is a less important molecule regarding these effects compared to n-butyrate [67]. On the other hand, isobutyrate has been described to promote the intestinal development of calves along with elevated mRNA expression of sodium–glucose cotransportes-1 (SGLT1), growth hormone receptors and insulin receptors [68].

According to our results, considering the proportion of various VFAs, lactulose had far more intense effects than psyllium. On day 5 of the experiment, lactulose increased the amount of propionate relative to the other VFAs. On day 10, lactulose supplementation further increased the propionate proportion, but acetate still accounted for more than half of the VFA content of the fecal samples. Its proportion had decreased compared to its initial concentration, but this change was not significant. On the last day of our study, lactulose significantly increased the concentration of propionate compared to the other VFAs. From the present results, it can be concluded that lactulose increased the concentration of propionate to the highest extent compared to the other VFAs on day 10 of the experiment. On day 15, acetate was still the most abundant of the VFAs, but there was no significant difference observed compared to its initial mol% concentration. The proportion of n-butyrate showed a gradually decreasing tendency on day 5, day 10 and day 15 of the lactulose treatment.

In summary, lactulose and psyllium treatment increased the total VFA, acetate and propionate content. Furthermore, psyllium also increased the n-butyrate concentration on day 15, and the two treatments modified the proportion of VFAs to each other in a different manner. According to our results, an increase in total VFA concentrations was observed in the lactulose-treated animals already on day 10, while a similar increase was found in the psyllium group, but only on day 15 of the experiment. Notwithstanding that the present study provided novel data on the effects of prebiotics on intestinal VFA production in healthy dogs, its limitations should be also stressed; hence, future studies are needed to measure further fecal parameters including consistency, dry matter, ammonia–nitrogen (NH3-N) and pH, and to analyze the suggested changes in the composition of the gut microbiota.

## 5. Conclusions

Based on our results, we can conclude that psyllium increased the total VFA, acetate and propionate content only on day 15 of the experiment. This finding suggests a potentially longer adaptation time for the microbiome to break down the complex polysaccharides of psyllium, compared to the rapid fermentation of disaccharide lactulose. On the other hand, since lactulose was a more potent molecule to swiftly increase the total VFA, acetate and propionate concentration, as well as resulted in the shift of microbiota in the direction of propionate-producing bacteria, it may be suggested that it alters more rapidly and intensely the intestinal microbiota.

Our results also draw attention to the fact that not only probiotics but also prebiotics can have significant effects in the gastrointestinal tract and that we should therefore increasingly aim at the use of dietary supplements with a conscious, targeted prebiotic effect. Prebiotics have been widely used in human medicine for decades and there has recently been increasing interest and demand in their use in veterinary medicine. There is currently little evidence as to whether prebiotics can be useful as adjunctive treatment for canine diseases such as intestinal tract infections, constipation, HE, PSS and certain pathologies of liver and kidney failure. Further research is required to explore the potential role of prebiotics in canine diseases and to link prebiotic-induced changes in the gut microbiota to significant physiological outcomes.

## Data Availability

All raw data supporting the results of the present study can be obtained from the corresponding author upon reasonable request.

## Supplementary Materials

The following supporting information can be downloaded at: https://www.mdpi.com/article/10.3390/vetsci9050206/s1, Figure S1: Violin plots show the concentration of isobutyrate in the faecal samples following lactulose (A) and psyllium (B) treatment measured by GC-MS method; Figure S2: Violin plots show the concentration of isovalerate in the faecal samples following lactulose (A) and psyllium (B) treatment measured by GC-MS method; Table S1: Age, sex and weight of the animals, involved in the study; Table S2: Haematological parameters of the dogs. The top row shows the reference values for healthy animals; Table S3: Biochemical parameters of the dogs. The top row shows the reference values for healthy animals.
