# Supplementary material for: Altered Intestinal Production of Volatile Fatty Acids in Dogs Triggered by Lactulose and Psyllium Treatment
Source: Vet Sci. 2022 Apr 23;9(5):206. doi: 10.3390/vetsci9050206 (PMC9145803; doi:10.3390/vetsci9050206)
Supplement: Supplementary file 1 [file vetsci-09-00206-s001.zip › vetsci-1653689-supplementary.pdf]

veterinary sciences

MDPI

Supplementary

Altered intestinal volatile fatty acid production in dogs triggered by lactulose and psyllium treatment

Máté Mackei [1*], Rebeka Talabér [1], Linda Müller [2], Ágnes Sterczer [3], Hedvig Fébel [4], Zsuzsanna Neogrády [1] and Gábor Mátis [1]

[1] Division of Biochemistry, Department of Physiology and Biochemistry, University of Veterinary Medicine, István street 2, H-1078 Budapest, Hungary
[2] Department of Obstetrics and Food Animal Medicine Clinic, University of Veterinary Medicine, István street 2, H-1078 Budapest, Hungary
[3] Department and Clinic of Internal Medicine, University of Veterinary Medicine, István street 2, H-1078 Budapest, Hungary
[4] Nutrition Physiology Research Group, Institute of Physiology and Nutrition, Kaposvár Campus, Hungarian University of Agriculture and Life Sciences, Gesztenyés street 1, H-2053 Herceghalom, Hungary
* Correspondence: mackei.mate@univet.hu

Animal ID	Sex	Body weight (kg)	Age (months)	Treatment
1	male	17.2	59	Lactulose
2	male	15.5	59	Lactulose
3	male	17.3	34	Lactulose
4	male	16	28	Lactulose
5	male	16.8	28	Lactulose
6	male	15.2	28	Lactulose
7	male	16.5	70	Lactulose
8	male	19	35	Lactulose
9	male	15.5	59	Lactulose
10	male	15.6	37	Lactulose
11	male	16.5	60	Psyllium
12	male	14.4	38	Psyllium
13	male	15.2	38	Psyllium
14	male	11.5	38	Psyllium
15	male	15.9	37	Psyllium
16	male	12.8	37	Psyllium
17	male	14.4	59	Psyllium
18	male	14.5	37	Psyllium
19	male	10.9	38	Psyllium
20	male	13.5	71	Psyllium
21	female	17	71	Lactulose
22	female	14.3	71	Lactulose

23	female	13.8	27	Lactulose
24	female	14.1	28	Lactulose
25	female	12.9	27	Lactulose
26	female	17.8	66	Psyllium
27	female	15.8	71	Psyllium
28	female	15.9	70	Psyllium
29	female	13	70	Psyllium
30	female	12	71	Psyllium

Table S1: Age, sex and weight of the animals, involved in the study

Animal ID	WBC [10^9/L]	NEU [10^9/L]	LYM [10^9/L]	MON [10^9/L]	EO [10^9/L]	BASO [10^9/L]	RBC [10^{12}/L]	HGB [g/L]	HCT [L/L]	MCV [fL]	MCH [pg]	PLT [10^9/L]
Reference values	**7.2-21.9**	**3.8-14.2**	**1.9-6.3**	**0.4-2.1**	**<2.45**	**<0.09**	**5.53-8.20**	**127-190**	**0.37-0.53**	**60.1-71.6**	**21.7-24.4**	**156-582**
1	17.37	8.34	4.74	1.63	2.61	0.05	7.06	154	0.42	59.3	21.8	396
2	10.85	6.11	3.63	0.77	0.32	0.02	7.4	175	0.49	65.8	23.6	259
3	11.45	5.6	4.45	0.54	0.85	0.01	7.94	191	0.52	65.2	24.1	243
4	11	5.86	3.56	0.82	0.74	0.02	7.74	173	0.47	60.5	22.4	298
5	12.29	7.4	3.12	0.81	0.93	0.03	7.37	174	0.48	64.5	23.6	298
6	11.43	6.1	4.30	0.58	0.42	0.03	7.97	189	0.50	63.1	23.7	324
7	10.46	5.92	2.96	0.89	0.68	0.01	6.72	156	0.44	65.0	23.2	411
8	10.01	4.99	4.01	0.74	0.25	0.02	7.68	187	0.49	64.2	24.3	283
9	11.27	7.13	3.01	0.77	0.33	0.03	7.08	168	0.46	64.4	23.7	345
10	14.93	9.67	4.05	0.6	0.59	0.02	7.17	170	0.46	64.4	23.7	397
11	9.21	5.39	2.55	0.52	0.73	0.02	7.79	189	0.51	65	24.3	195
12	10.86	7.79	2.07	0.55	0.44	0.01	6.6	158	0.43	65.6	23.9	360
13	19.46	12.63	4.06	1.4	1.33	0.04	6.24	148	0.4	64.6	23.7	469
14	12.78	6.61	3.25	0.96	1.93	0.03	7.05	166	0.44	62.6	23.5	304
15	12.95	8.84	2.7	0.75	0.64	0.02	6.55	168	0.46	70.5	25.6	377
16	12.55	7.73	2.47	0.55	1.77	0.03	7.77	181	0.48	61.4	23.3	336
17	18.4	14.15	2.87	0.84	0.53	0.01	7.13	174	0.46	65.1	24.4	445
18	15.36	11.28	2.81	1.06	0.19	0.02	7.82	181	0.48	60.9	23.1	432
19	11.72	7.7	2.75	0.81	0.4	0.06	7.01	166	0.45	63.5	23.7	437
20	10.35	6.05	2.52	0.79	0.94	0.05	7.06	168	0.47	66.9	23.8	554
21	12.47	7.46	3.85	0.67	0.48	0.01	7.05	157	0.43	61.3	22.3	491
22	11.41	6.31	4.24	0.65	0.18	0.03	8.6	193	0.51	59.2	22.4	407
23	11.62	6.69	4.01	0.59	0.3	0.03	7.19	170	0.46	63.7	23.6	302
24	15.42	7.28	4.76	0.86	2.49	0.03	7	164	0.44	63.3	23.4	438
25	14.7	8.02	3.29	0.54	2.83	0.02	7.62	175	0.48	62.6	23	325
26	9.88	5.9	2.42	0.57	0.98	0.01	7.11	168	0.46	64.8	23.6	314
27	9.33	6.44	2.07	0.4	0.41	0.01	8.41	197	0.54	63.9	23.4	432
28	9.51	5.76	1.82	0.42	1.5	0.01	8.78	210	0.57	65.4	23.9	260

29	8.01	5.36	1.93	0.3	0.41	0.01	6.64	153	0.42	62.5	23	458
30	11.39	6.83	3.13	0.64	0.77	0.02	7.11	163	0.44	61.5	22.9	441

Table S2. Haematological parameters of the dogs. The top row shows the reference values for healthy animals. WBC= white blood cells, NEU= neutrophil granulocytes, LYM= lymphocytes, MON= monocytes, EO= eosinophil granulocytes, BASO= basophil granulocytes, RBC= red blood cells, HGB= haemoglobin, HCT= haematocrit, MCV= mean red cell volume, MCH= mean haemoglobin content of red cells, PLT= platelets.

Animal ID	ALT [IU/L]	LIP [IU/L]	GGT [IU/L]	TG [mmol/L]	CREA [µmol/L]	ALB [g/L]	TPROT [g/L]
**Reference values**	23-147	<150	2.3-9.1	0.18-0.89	33.5-76.3	27-35	50-65
**1**	23.4	70	4	0.48	70	27.6	57.8
**2**	33.9	154	5.3	0.84	48	30.6	60.7
**3**	51.6	26	4	0.77	81.5	32.6	61.5
**4**	43.4	41	4.4	0.68	61.1	32.9	59.6
**5**	64.5	42	3	0.7	53.5	33	60.2
**6**	42.1	65	4.6	0.75	64.5	31.1	58.9
**7**	39	30	3.6	0.83	59.4	28.6	66.7
**8**	45.1	71	6.1	0.78	65.8	33.7	58.6
**9**	35.3	121	4.6	0.61	48.2	30.4	57.4
**10**	33	76	4.5	0.42	66.4	31.2	57.5
**11**	132.6	37	3.5	0.43	65.7	33.3	61.6
**12**	54.6	54	4.6	0.64	57.7	29.9	57.2
**13**	164.5	46	3.3	0.5	53.8	30.7	56.2
**14**	48.9	93	4.8	0.31	51.7	31.1	58.3
**15**	51	26	3.4	0.41	68.2	32.6	60.9
**16**	99	58	3.8	0.49	61.1	32.5	63.1
**17**	63.8	32	2.4	0.41	40.2	33.3	62
**18**	52	36	2.6	0.47	57.1	33.1	60.5
**19**	31.2	46	5.1	0.44	59.9	32.3	56.3
**20**	61.7	69	2.5	0.5	49	30.8	59.1
**21**	25.6	111	4.7	0.76	46.8	32.7	60
**22**	38.4	141	4	0.63	43.9	33.9	66
**23**	36.1	93	3.3	0.55	39.8	33.8	60
**24**	62.6	97	4.3	0.54	47.4	31.6	57.8
**25**	34.9	52	3.8	0.56	53.9	32.4	56.2
**26**	44.5	82	5.2	0.62	48.8	29.7	61.7
**27**	80.6	52	5.7	0.83	56.7	34	66
**28**	84	127	3.2	0.48	41.9	31.8	62.4
**29**	56	59	2.9	0.58	57.2	32	61.3
**30**	70.5	95	2.9	0.77	50.4	31.7	63.4

Table S3: Biochemical parameters of the dogs. The top row shows the reference values for healthy animals. ALT= alanine aminotransferase, LIP= lipase, GGT= gamma-glutamyl transferase, TG= triglyceride, CREA= creatinine, ALB= albumin, TPROT= total protein

Figure S1. Violin plots show the concentration of isobutyrate in the faecal samples following lactulose **(A)** and psyllium **(B)** treatment measured by GC-MS method. Data have been visualized using violin plots. Concentration of single samples are plotted as grey dots; black line refers to the median and grey lines refer to first and third quartiles.

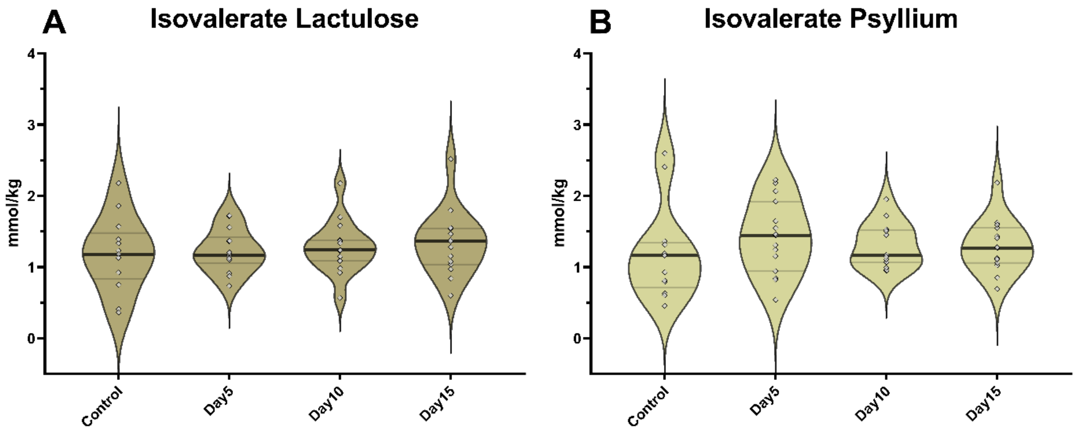

Figure S2. Violin plots show the concentration of isovalerate in the faecal samples following lactulose **(A)** and psyllium **(B)** treatment measured by GC-MS method. Data have been visualized using violin plots. Concentration of single samples are plotted as grey dots; black line refers to the median and grey lines refer to first and third quartiles.

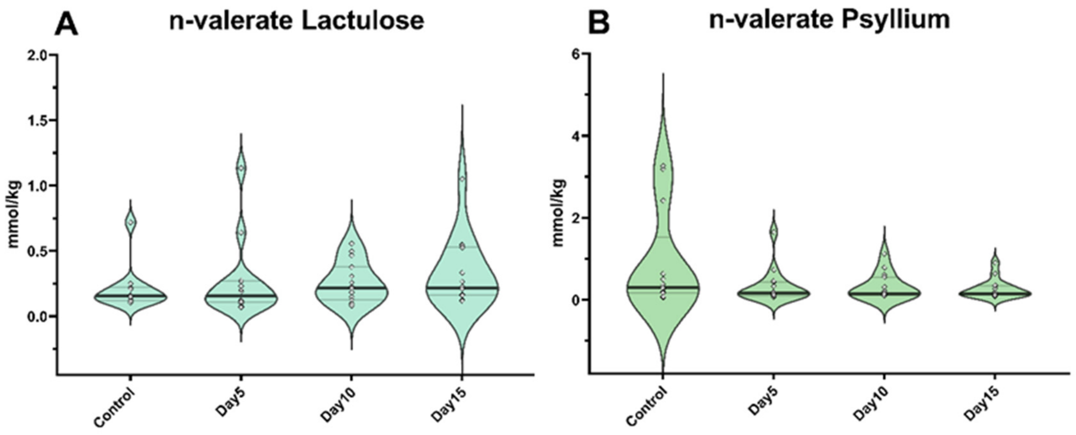

Figure S3. Violin plots show the concentration of n-valerate in the faecal samples following lactulose **(A)** and psyllium **(B)** treatment measured by GC-MS method. Data have been visualized using violin plots. Concentration of single samples are plotted as grey dots; black line refers to the median and lines refer to first and third quartiles.
